# Whole-Genome Sequences of Two Kenyan Aspergillus minisclerotigenes Strains

**DOI:** 10.1128/mra.00219-23

**Published:** 2023-07-05

**Authors:** Alexandra Schamann, Rolf Geisen, Markus Schmidt-Heydt

**Affiliations:** a Max Rubner-Institut, Federal Research Institute of Nutrition and Food, Department of Safety and Quality of Fruit and Vegetables, Karlsruhe, Germany; Vanderbilt University

## Abstract

Here, we report the sequencing of the whole genome, including the mitochondrial DNA, of the two highly aflatoxigenic Aspergillus minisclerotigenes strains MRI390 and MRI400 using the MiSeq and PacBio platforms and the generated assemblies. The strains were isolated from Kenyan maize kernels.

## ANNOUNCEMENT

Aspergillus minisclerotigenes is a highly aflatoxigenic Aspergillus section *Flavi* species repeatedly isolated from samples originating from Kenyan regions, where aflatoxin contamination poses an exceptional risk to food safety and several outbreaks of aflatoxicosis have been reported (1–3). Nevertheless, limited research has focused on *A. minisclerotigenes* until now. To increase our knowledge of this fungus, the genomes of the Kenyan *A. minisclerotigenes* strains MRI390 and MRI400 were sequenced using the MiSeq and PacBio platforms.

**FIG 1:**
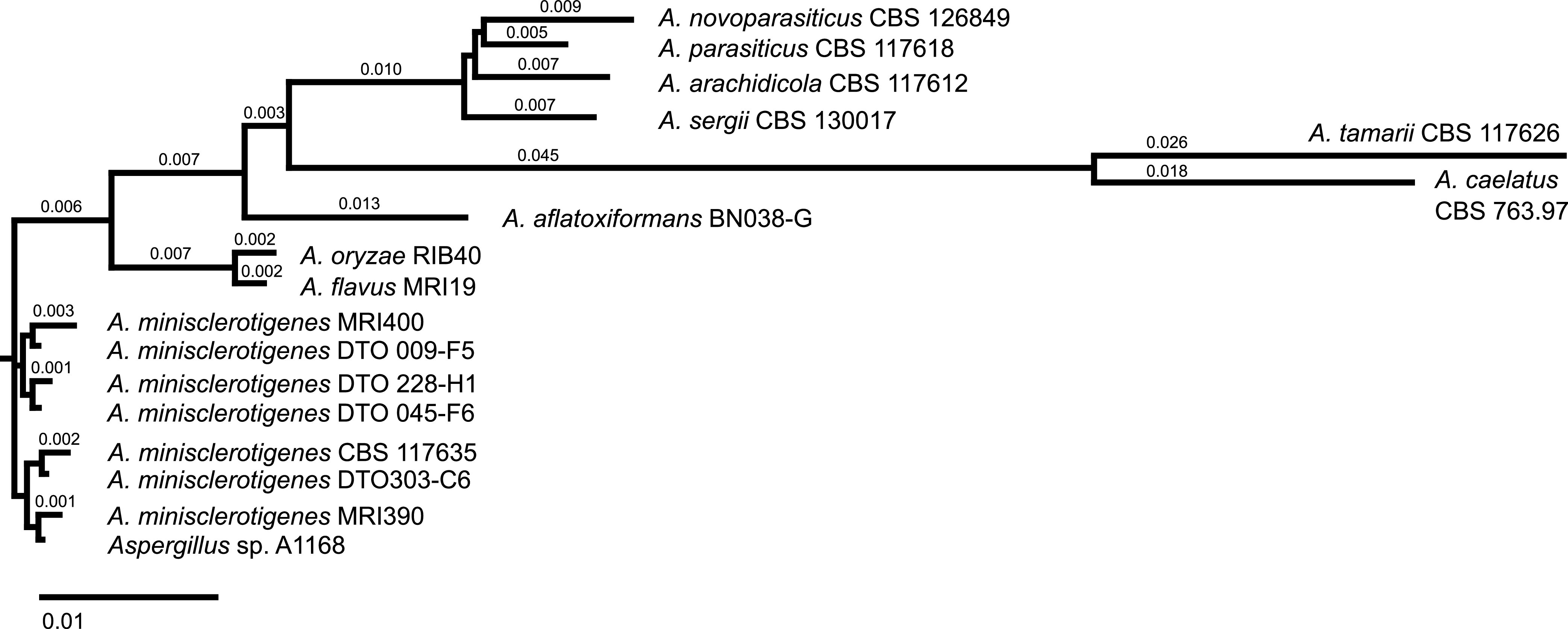
Phylogenetic tree of various *Aspergillus* strains, based on partial sequencing of the β-tubulin, calmodulin, and nitrate reductase genes.

The strains were isolated from ground maize kernels from Katumani, Kenya, by mixing the ground kernels with a Tween 80-NaCl solution (9 g/L NaCl, 1 g/L Tween 80, 1 g/L agar), generating a dilution series, and cultivating the dilutions on selective nutrient medium. The isolates were identified by partial sequencing of the β-tubulin (Bt2a/2b [4]), calmodulin (cmd5/6 [5], cmd2F/2R [6]), and nitrate reductase (niaDF/AR [6], niaDBF/BR [6, 7], niaDCF/CR [8]) genes. For this, PCR was performed using the peqGOLD Taq DNA polymerase all-inclusive kit (VWR International GmbH, Darmstadt, Germany) with 2.5 μL of each primer (5 pmol/μL) and 5 μL DNA. Amplification was achieved with the following cycling program: 95°C for 3 min; 40 cycles of 95°C for 30 s, 52°C (cmd2F/2R)/55°C (niaDF/AR)/57°C (niaDBF/BR, niaDCF/CR)/60°C (Bt2a/2b, cmd5/6) for 40 s, and 72°C for 90 s; and 72°C for 3 min. The PCR products were sequenced in both directions by Eurofins Genomics (Cologne, Germany) using Sanger technology, and the sequences were assembled (SeqMan Pro, LaserGene v17). The two respectively three overlapping consensus sequences of calmodulin respectively nitrate reductase were concatenated (MegAlign Pro, SeqBuilder Pro). The sequences of the three partial genes (β-tubulin, calmodulin, nitrate reductase) were compared to sequences in NCBI using BLASTN. Concatenating these three gene sequences, a phylogenetic tree was created using the neighbor-joining algorithm with the same partial genes of a variety of different Aspergillus strains: Aspergillus aflatoxiformans BN038-G (GenBank accession no. MK119747.1, MK119713.1, MK119679.1) (7), Aspergillus arachidicola CBS 117612 (ML737115.1, ML737234.1, ML737155.1) (9), Aspergillus caelatus CBS 763.97 (NW_022475357.1, NW_022475408.1, NW_022475603.1) (9), Aspergillus flavus MRI19 (JAGYXF010000057.1, JAGYXF010000047.1, JAGYXF010000013.1) (10), *A. minisclerotigenes* CBS 117635 (ML732812.1, ML732765.1, ML732764.1) (9), *A. minisclerotigenes* DTO 009-F5 (MT024508.1, MT024497.1, MT024519.1) (11), *A. minisclerotigenes* DTO 228-H1 (MT024515.1, MT024504.1, MT024526.1) (11), *A. minisclerotigenes* DTO 045-F6 (MT024512.1, MT024501.1, MT024523.1) (11), *A. minisclerotigenes* DTO 303-C6 (MT024516.1, MT024506.1, MT024528.1) (11), Aspergillus novoparasiticus CBS 126849 (ML733430.1, ML733443.1, ML733467.1) (9), Aspergillus oryzae RIB40 (NC_036440.1, NC_036436.1, NC_036438.1) (12), Aspergillus parasiticus CBS 117618 (ML734942.1, ML734938.1, ML734939.1) (9), Aspergillus sergii CBS 130017 (ML741807.1, ML741799.1, ML741762.1) (9), Aspergillus tamarii CBS 117626 (ML738700.1, ML738591.1, ML738590.1) (9), and Aspergillus sp. strain A1168 (MK119750.1, MN987082.1, MK119682.1) (7, 13) (Fig. 1). The identification was confirmed by the experts at the Westerdijk Fungal Biodiversity Institute (Utrecht, Netherlands). For DNA extraction, the fungal strains were grown for 4 days on potato-dextrose agar at 25°C, and the mycelium was homogenized using liquid nitrogen and a mortar and pestle. For MiSeq sequencing, DNA was extracted using the NucleoSpin plant II kit (Macherey-Nagel, Düren, Germany) following the manufacturer’s instructions. For PacBio sequencing, DNA of the homogenized mycelium was extracted using cetyltrimethylammonium bromide (CTAB) buffer (0.1 M Tris [pH 8.0], 1.4 M NaCl, 20 mM EDTA, 2% [wt/vol] CTAB, 4% [wt/vol] polyvinylpyrrolidone having an average molecular weight of 10,000 [PVP-10]) for lysis, phenol-chloroform for extraction, and propan-2-ol and 7.5 M ammonium acetate for precipitation overnight. Two DNA extraction protocols were followed due to the different quality and quantity requirements of the DNA, which were checked using a NanoDrop 1000 spectrophotometer and a Qubit 3.0 fluorometer (both from Thermo Fisher Scientific GmbH, Bremen, Germany).

A library was prepared using the Illumina DNA prep kit (San Diego, USA) and sequenced (2 × 300 bp) on the MiSeq platform (Illumina). The raw reads were quality checked (FastQC v0.11.3), as well as quality trimmed and checked for remaining adapter sequences (Trimmomatic v0.39) (14). A PacBio sequencing library was prepared using the SMRTbell Express template prep kit 2.0 (Pacific BioSciences, Menlo Park, USA) following the manufacturer’s instructions. DNA was sheared into fragments of 6 to 10 kb using g-TUBE devices (Covaris, Brighton, UK). BluePippin (Sage Science, Beverly, USA) was used for size selection, before sequencing on the Sequel platform by BGI (Hong Kong, China). The quality of the PacBio data was checked using LongQC v1.2.0 (15).

Default software parameters were used except where otherwise noted. Different assembly tools were tested for each strain, and the software fitting best to each was chosen for the final assembly. Combining the data from both technologies, *de novo* hybrid assembly was carried out using SPAdes v3.14.1 for strain MRI400 (16, 17). For strain MRI390, *de novo* assembly of the PacBio data was performed using Flye v2.8.2, and then the MiSeq data were aligned to the resulting contigs; the alignment was polished using Pilon v1.23 and SAMtools v1.10 (17–19). Short contigs (<400 bp) were excluded. In addition to the genomic DNA, the complete mitochondrial DNA was sequenced. The completeness of the genome assemblies was determined using BUSCO v5.4.6 with the lineage database ascomycota_odb10 (20). The sequencing data and assembly metrics are shown in Table 1. The genomes of *A. minisclerotigenes* will be analyzed more deeply and compared to other aflatoxigenic Aspergillus strains, focusing especially on the aflatoxin gene cluster.

**TABLE 1:** Sequencing and assembly data

Parameter	Data for Aspergillus minisclerotigenes strain:	
	MRI390	MRI400
Genome size (Mb)	38.03	37.88
Mitochondrial DNA (bp)	29,195	29,329
No. of contigs	28	52
GC content (%)	47.51	47.51
*N*_50_ value (bp)	3,823,310	1,345,410
Coverage (×)	74	102
Total no. of MiSeq paired-end raw reads	28,506,465	31,129,593
PacBio data		
Total no. of raw reads	288,480	161,135
*N*_50_ value (bp)	11,239	10,403
Avg read length (bp)	9,676	9,105
Total no. of BUSCO orthologs	1,706	1,706
Complete single-copy, complete multicopy, fragmented, and missing orthologs (%)	93.7, 0.9, 0.5, 4.9	93.4, 0.8, 0.6, 5.2

### Data availability.

This whole-genome shotgun project has been deposited at DDBJ/ENA/GenBank under accession no. JAHXGP000000000 and JAHYSD000000000 and BioProject accession no. PRJNA742918 and PRJNA741918. The versions described in this paper are versions JAHXGP010000000 and JAHYSD010000000. The raw sequence reads have been deposited in the Sequence Read Archive (SRA) under accession no. SRR15400241, SRR15400240, SRR15130309, and SRR15130308. The partial gene sequences have been deposited at GenBank under accession no. OQ909815, OQ909818, OQ909821, OQ909816, OQ909819, and OQ909822.
